# Future projections of the prevalence of dementia in Japan: results from the Toyama Dementia Survey

**DOI:** 10.1186/s12877-021-02540-z

**Published:** 2021-10-26

**Authors:** Nobue Nakahori, Michikazu Sekine, Masaaki Yamada, Takashi Tatsuse, Hideki Kido, Michio Suzuki

**Affiliations:** 1 Faculty of Nursing Science, Tsuruga Nursing University, 78-2-1 Kizaki, Tsuruga, Fukui 914-0814 Japan; 2 Department of Epidemiology and Health Policy, School of Medicine, University of Toyama, 2630 Sugitani, Toyama, Toyama 930-0194 Japan; 3 Kiseikai, Kido Clinic, 244 Honoki, Imizu, Toyama 934-0053 Japan; 4 Department of Neuropsychiatry, School of Medicine, University of Toyama, 2630 Sugitani, Toyama, Toyama 930-0194 Japan

**Keywords:** Dementia, Future projections, Japan, Prevalence

## Abstract

**Background:**

This study aimed to make future projections of the nationwide prevalence of dementia in Japan using the prevalence of dementia from the Toyama Dementia Survey and population projections.

**Methods:**

We performed linear regression analysis using the prevalence of dementia by sex and age in 1985, 1990, 1996, 2001, and 2014 from the Toyama Dementia Survey to calculate the estimated future prevalence by sex and age. The estimated prevalence was then multiplied by the estimated future population of people aged 65 years and older by sex and age in each of the 47 prefectures from 2020 to 2045 and added together to calculate the total number of people with dementia. The estimated future prevalence of dementia was calculated by dividing the calculated number of people with dementia by the estimated future population of people aged 65 years and older in each of the 47 prefectures. In addition, the estimated future prevalence of dementia in each of the 47 prefectures from 2020 to 2045 was presented on a map of Japan and grayscale-coded in four levels.

**Results:**

In 2020, the estimated future prevalence of dementia did not exceed 20% in any prefecture, but in 2025, five prefectures, mainly rural prefectures, had projected rates exceeding 20%. In 2030, the prevalence rate is projected to exceed 20% nationwide, and by 2035, the rate will exceed 25% in 42 prefectures. In 2045, all prefectures excluding Tokyo are projected to have a dementia prevalence rate exceeding 25%, and the rate will exceed 30% in 12 of 47 prefectures.

**Conclusions:**

Over the next 25 years, the prevalence of dementia in people older than 65 years is projected to exceed 25% nationwide, including metropolitan areas.

## Background

In Japan, the prevalence of dementia is critically increasing with population aging. Regarding the future projections of dementia, a group at Kyushu University estimated the future prevalence of elderly people with dementia in Japan based on data from all investigations conducted in Hisayama, Fukuoka Prefecture [1], and it is predicted that one of five people older than 65 will have dementia by 2025 [2]. A group at the University of Toyama also examined the current status of dementia in Toyama Prefecture based on data from five surveys of elderly people with dementia in Toyama Prefecture since 1985. Using the future population projections published by the National Institute of Population and Social Security Research [3, 4], the Toyama group projected prevalence of dementia in Toyama prefecture, through 2045 [5, 6]. In this context, if the prevalence of dementia continues to grow linearly, the rate of dementia in the elderly in 2045 would be 1 in 3.5. However, if we assume that the prevalence rate in the 2014 survey remains constant, one in every 5.4 people aged 65 and older will have dementia, which is a large difference of approximately 10% from the prevalence rate if it grows linearly to 2045. To prevent the gap from widening, we must implement preventive measures to delay the onset of dementia as much as possible to slow the acceleration of its prevalence.

In addition, when we examine the problem of the increased prevalence of dementia in Japan based on data from Toyama Prefecture, in which the aging rate is slightly higher than the national average, we can predict that the same situation in Toyama Prefecture will eventually occur throughout the country. Visualizing this reality may provide an opportunity to consider countermeasures. However, there are no studies that have predicted future projections in the prevalence of dementia in Japan by prefecture using a highly accurate prediction model.

Therefore, the purpose of this study was to predict the future prevalence of dementia in each prefecture using the prevalence of dementia from the actual survey in Toyama Prefecture and the estimated future population of each prefecture.

## Methods

### Participants

The Toyama Dementia Survey assessed the prevalence of dementia among randomly selected elderly people aged 65 years and older who resided in Toyama Prefecture. Toyama Prefecture is located in the central region of Japan on the Sea of Japan side. The prefecture had a population of approximately 1 million as of 2020 (37th among the 47 prefectures), a population density of 243 people/km^2^ (25th), and an aging population rate of 32.7% (13th), making it a rural prefecture with an aging population. The survey has been conducted six times to date (1983, 1985, 1990, 1996, 2001, and 2014). The primary survey is conducted by public health nurses using the Revised Hasegawa Dementia Scale (HDS-R), and the secondary survey is conducted by interviewing people suspected of having dementia based on their HDS-R scores and medical history. In this study, the results of the surveys conducted since 1985 were included in the analysis because psychiatrists have conducted the diagnosis of dementia since 1985, and the accuracy of the diagnosis is considered high.

The 2014 survey included 1537 randomly selected people (including institutional residents) 0.5% from 307,582 elderly people aged 65 years or older living in Toyama Prefecture as of October 1, 2013 (representing 0.5% of the population). The purpose of the study was explained by telephone to the selected subjects or their proxies, such as family members or facility staff, and they were asked to cooperate in the study. Of these, 1303 people agreed to participate in the study (consent rate: 84.8%). In previous studies conducted from 1985 to 2001, a survey request was made by randomly sampling 0.9–1% of elderly people aged 65 years or older in Toyama Prefecture, and the survey included 1416–2046 people who gave consent (consent rate: 90.0–96.8%). The University of Toyama Ethical Review Committee approved this study.

### Measurement

Age, gender, the HDS-R score, and the presence of dementia were investigated for each subject.

### Dementia diagnosis

The diagnosis of dementia was made using a two-stage survey method, the details of which have been published in other literature [7]. To summarize, in the first stage of the study, public health nurses conducted a home visit survey, which included the HDS-R. The HDS-R is a nine-item questionnaire for dementia screening with a maximum score of 30 points. The HDS-R is widely used in Japan, and it is recognized as valid based on its sensitivity and specific of 0.90 and 0.82, respectively (cutoff: 20/21) [8]. It is strongly correlated with the Mini-Mental State Examination [9]. Participants who scored less than 20 points on the HDS-R in the first-stage survey, those who reported a history of dementia, and those who were judged to require further investigation by the screening committee proceeded to the second-stage survey. In this survey, psychiatrists and municipal officials conducted home visits to diagnose dementia according to the International Classification of Diseases, Tenth Revision.

### Statistical analysis

The estimated future prevalence of dementia by sex and age group in Toyama Prefecture was based on previously reported data [6]. The estimated prevalence calculated via the linear regression equation (y = linear model coefficient × years + intercept) using the prevalence of dementia by sex and age group in the past five surveys from 1985 to 2014 was compared with the actual measured values. Pearson’s correlation coefficient was 0.98, indicating a strong correlation between the actual and estimated values.

Next, the prevalence of dementia by sex and age group obtained from the linear regression analysis was multiplied by the estimated future population by age in each of the 47 prefectures from 2020 to 2045 and then summed to calculate the total number of people with dementia. The prevalence of dementia was calculated by dividing the calculated number of people with dementia by the estimated future population by age in each prefecture. Future estimates of the prevalence of dementia in each prefecture from 2020 to 2045 were presented on a map of Japan and grayscale-coded at four levels.

SPSS ver. 23 was used for all analyses, and the significance level was set at less than 5%.

## Results

Table 1 presents an overview of the last five surveys of elderly people with dementia in Toyama Prefecture. The overall prevalence of dementia was 4.7% in 1985, versus 15.7% in 2014, representing an almost 3-fold increase. The overall age-adjusted prevalence rate also nearly doubled from 4.9% in 1985 to 9.6% in 2014. Regarding the prevalence of dementia by sex and age group, the prevalence of dementia in both men and women aged 80–84 and ≥ 85 years has been increasing since 1996. Among people aged 85 years and older in 2014, the prevalence was 38.3% in men and 47.4% in women.

**Table 1:** Summary of the Toyama Dementia Survey

	Year of survey				
	1985	1990	1996	2001	2014
Extraction rate (%)	1.0	0.9	1.0	1.0	0.5
Agreement rate (%)	94.4	96.8	92.2	90.0	84.8
Primary research collaborators	1416	1452	1,44	2046	1303
Second research collaborators	116	93	132	207	180
Dementia	60	73	102	159	146
Prevalence (%)	4.7	5.7	7.2	8.8	15.7
Age-adjusted disease rate (%)	4.9	5.4	5.7	7.0	9.6
Sex and age group disease rate (%)					
Male					
65–69	1.7	2.6	2.1	1.5	5.1
70–74	1.1	2.3	3.6	3.4	6.0
75–79	4.0	8.0	10.2	9.6	9.5
80–84	7.2	19.1	10.9	17.0	29.0
≥ 8590	11.1	21.2	14.4	22.6	38.3
Female					
65–69	0.9	1.5	0.3	3.0	1.3
70–74	1.7	1.4	1.8	2.8	3.6
75–79	6.3	2.2	4.2	6.1	8.1
80–84	21.5	10.5	13.5	15.5	22.8
≥ 8590	22.6	30.8	36.1	37.8	47.4

**Fig. 1:**
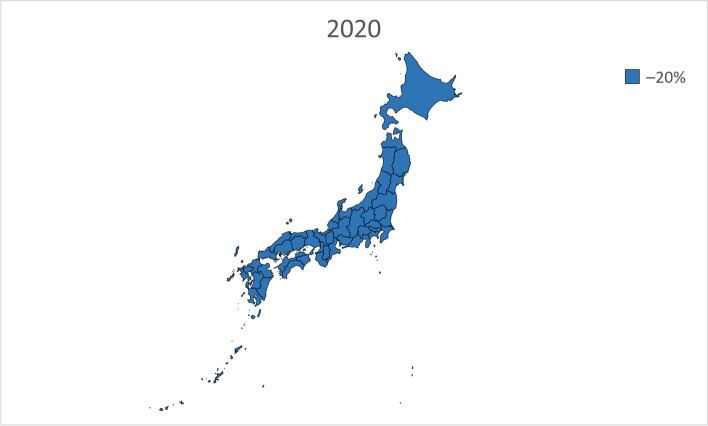
Estimation of the nationwide prevalence of dementia in 2020 via linear regression analysis

**Fig. 2:**
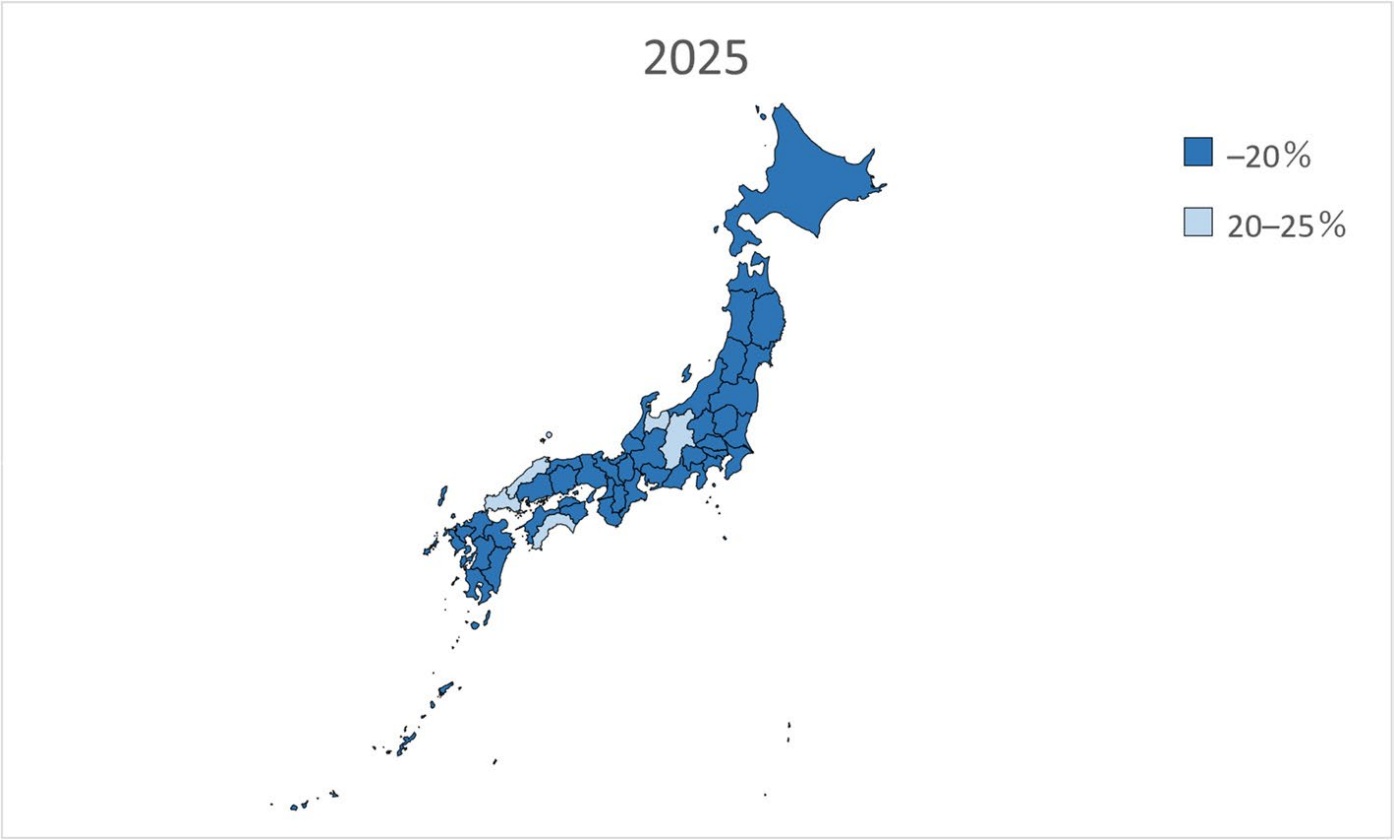
Estimation of the nationwide prevalence of dementia in 2025 via linear regression analysis

**Fig. 3:**
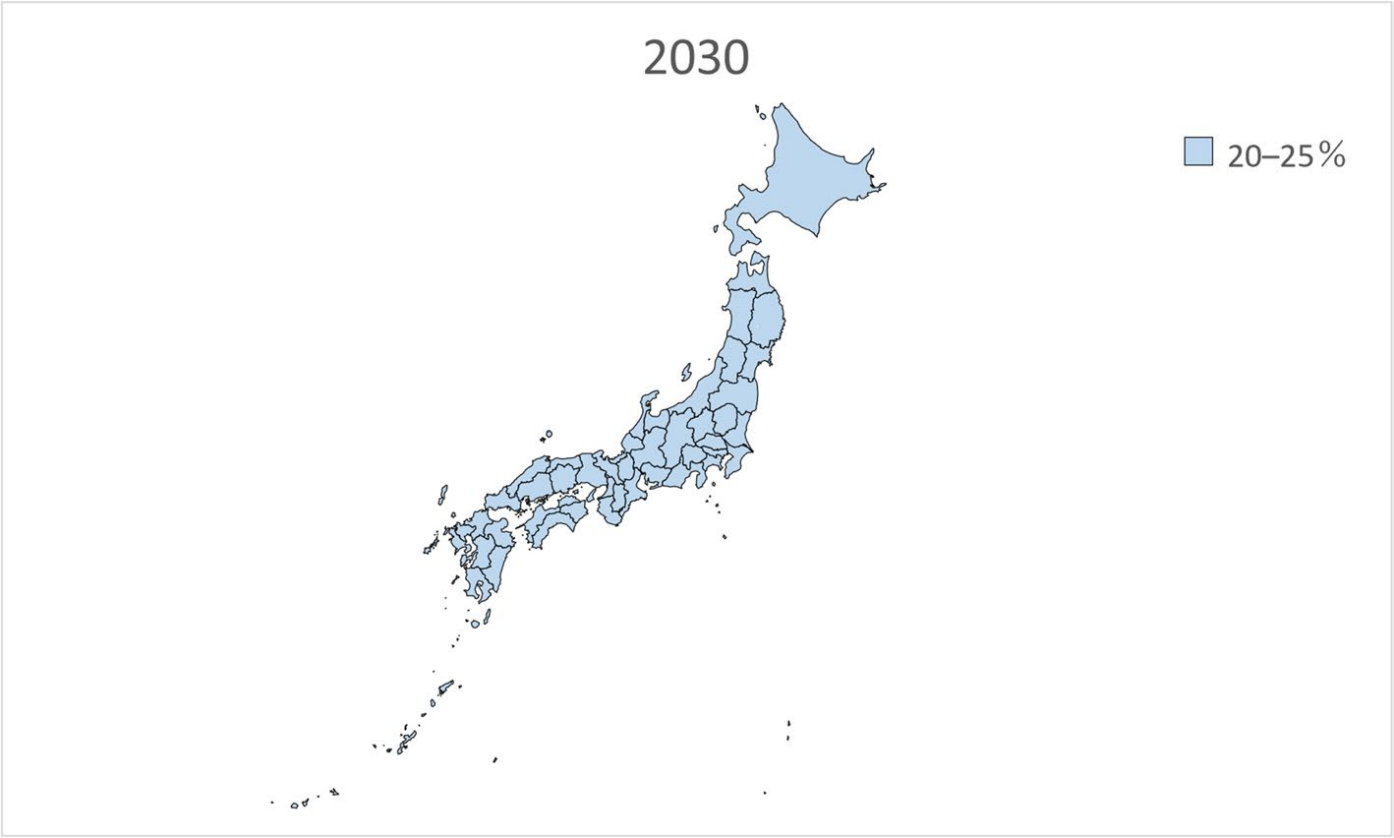
Estimation of the nationwide prevalence of dementia in 2030 via linear regression analysis

**Fig. 4:**
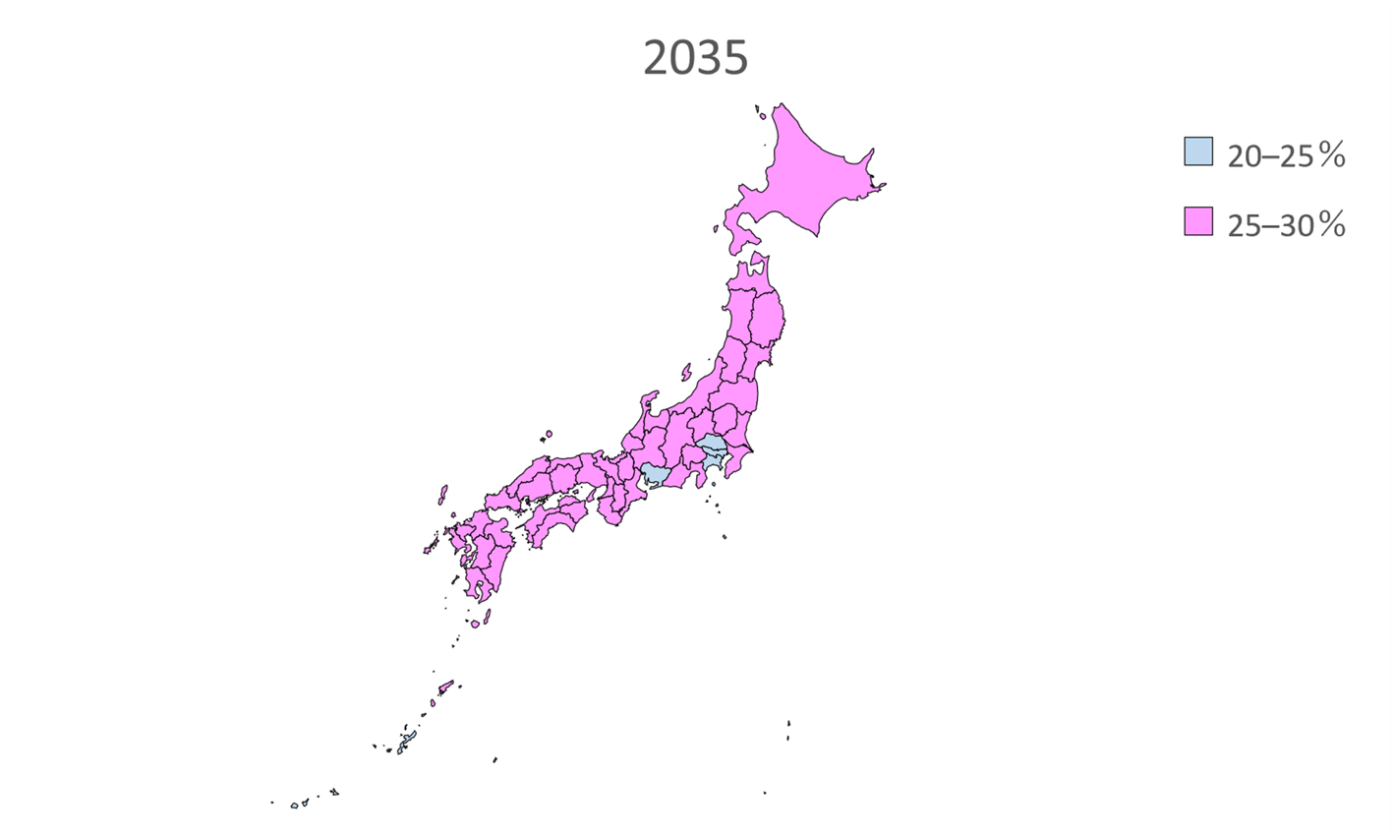
Estimation of the nationwide prevalence of dementia in 2035 via linear regression analysis

**Fig. 5:**
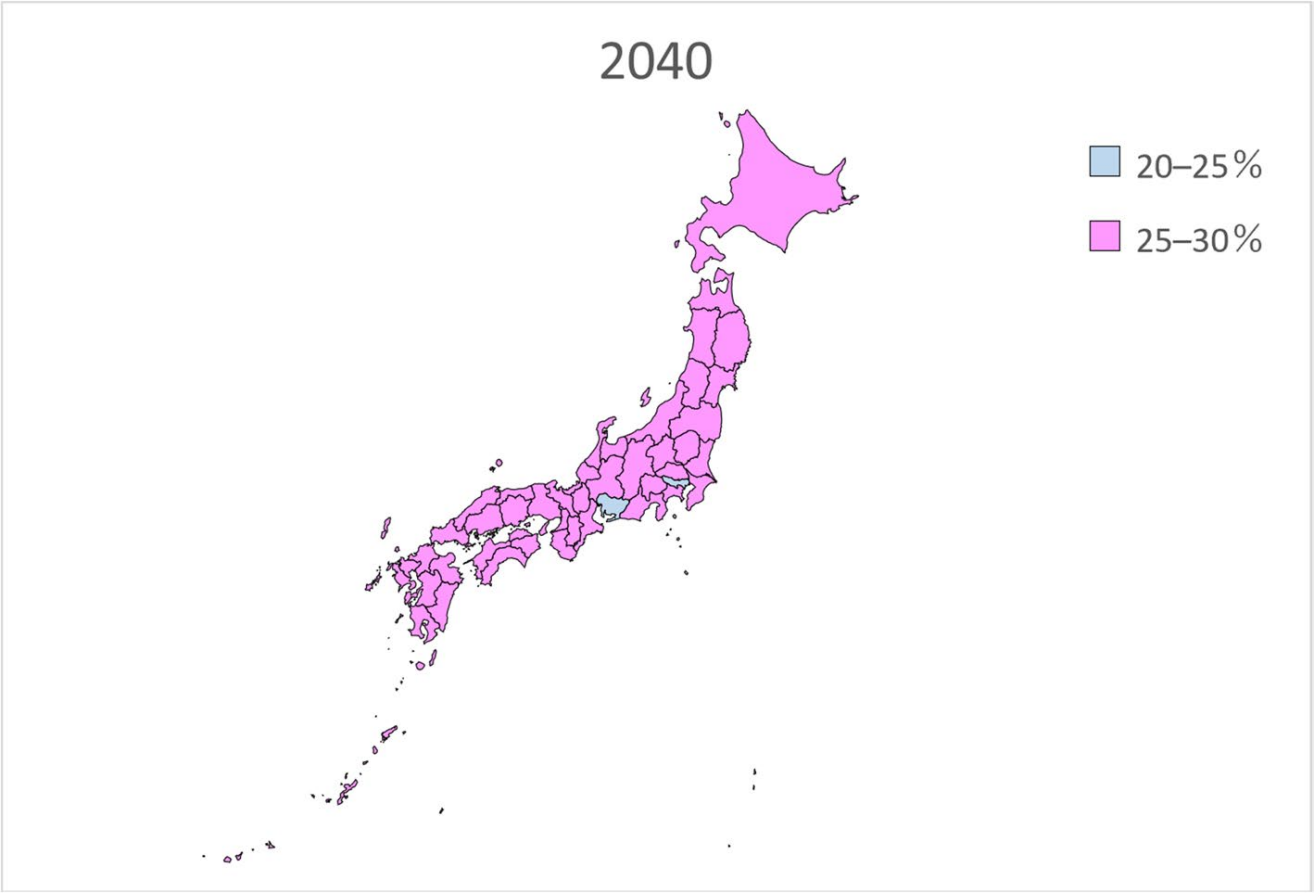
Estimation of the nationwide prevalence of dementia in 2040 via linear regression analysis

**Fig. 6:**
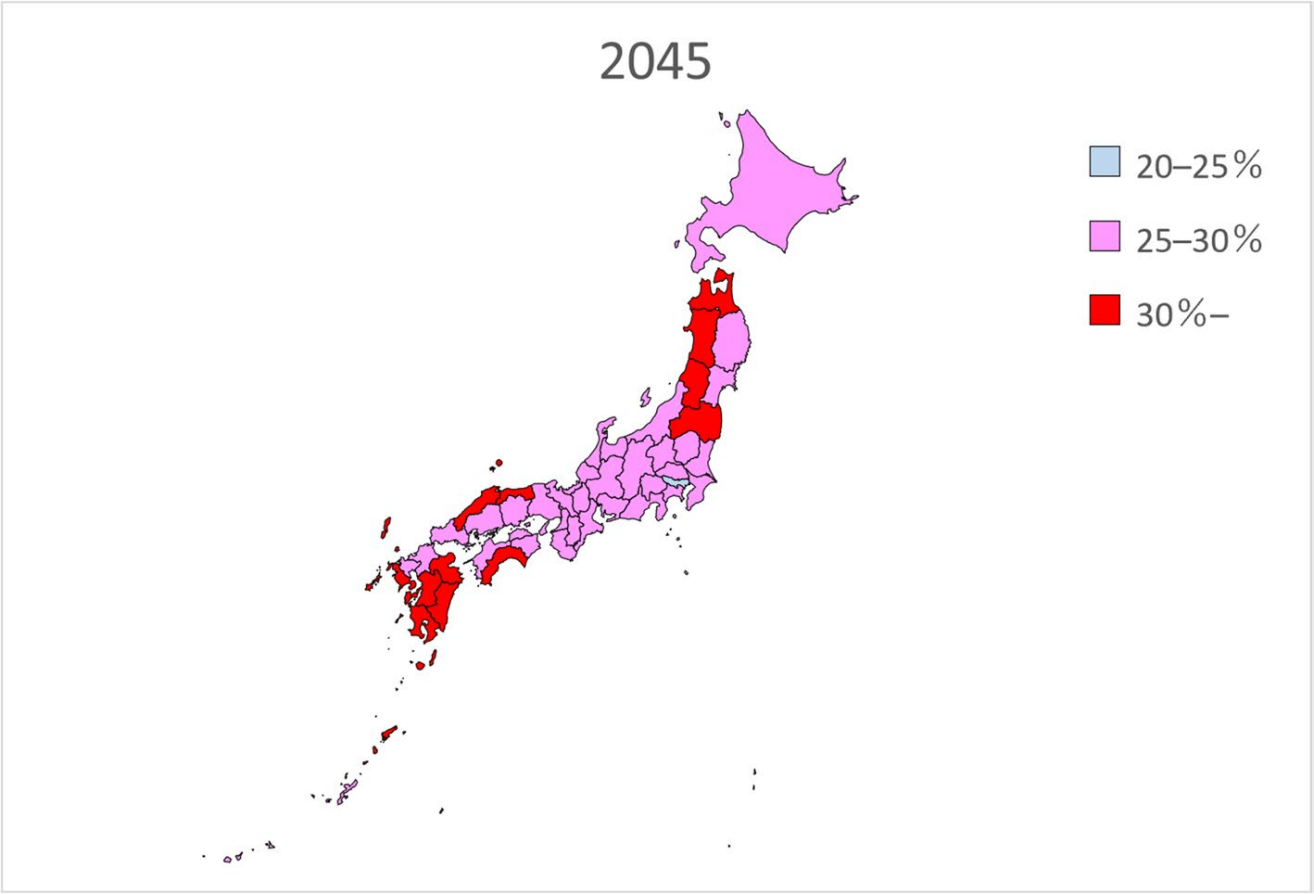
Estimation of the nationwide prevalence of dementia in 2045 via linear regression analysis

Table 2 presents the estimated future prevalence of dementia based on linear regression analysis for the entire country in 2020, 2025, 2030, 2035, 2040, and 2045. Figures 1, 2, 3, 4, 5 and 6 present the future estimated nationwide prevalence of dementia based on linear regression analysis in 2020, 2025, 2030, 2035, 2040, and 2045, respectively, at four levels (< 20, 20–25%, 25–30%, and > 30%) on a map.

**Table 2:** Future estimated nationwide prevalence of dementia according to linear regression analysis (%)

	2020	2025	2030	2035	2040	2045
Hokkaido	16.4	19.4	23.0	26.8	28.4	29.6
Aomori	16.5	18.9	22.0	25.7	28.2	30.1
Iwate	17.3	19.5	22.2	25.7	28.0	29.5
Miyagi	16.3	18.7	21.8	25.4	27.4	28.8
Akita	17.7	19.8	22.8	27.0	29.9	31.7
Yamagata	17.9	19.8	22.6	26.4	29.1	30.7
Fukushima	17.0	19.1	22.0	25.8	28.5	30.5
Ibaraki	15.5	18.4	22.1	25.6	27.0	28.3
Tochigi	15.5	18.0	21.6	25.2	26.7	27.9
Gunma	16.1	19.1	22.8	26.0	27.0	28.2
Saitama	14.7	18.5	22.4	24.8	25.0	26.1
Chiba	15.1	18.8	22.7	25.3	25.5	26.6
Tokyo	16.2	19.4	22.0	23.4	23.4	24.7
Kanagawa	15.8	19.5	22.8	24.8	25.1	26.5
Niigata	17.3	19.8	23.0	26.6	28.5	29.7
Toyama	16.9	20.0	23.9	27.3	27.6	28.4
Ishikawa	16.4	19.3	23.1	26.5	27.0	28.0
Fukui	17.2	19.6	22.8	26.2	27.6	29.0
Yamanashi	17.0	19.6	22.6	25.6	27.4	29.5
Nagano	17.8	20.5	23.8	26.7	27.8	29.2
Gifu	16.3	19.4	22.8	25.9	26.7	28.0
Shizuoka	16.4	19.5	23.1	26.1	27.3	28.8
Aichi	15.5	19.0	22.4	24.7	24.8	26.0
Mie	16.5	19.4	22.6	25.4	26.2	27.7
Shiga	15.9	18.7	22.1	25.2	25.9	27.0
Kyoto	16.3	19.9	23.8	26.7	26.7	27.7
Osaka	15.7	19.7	23.4	25.5	25.2	26.3
Hyogo	16.0	19.3	23.0	25.7	26.2	27.6
Nara	15.9	19.4	23.4	26.6	27.5	28.9
Wakayama	16.9	19.6	22.8	25.8	26.9	28.4
Tottori	17.7	19.9	23.1	27.0	28.9	30.1
Shimane	18.3	20.5	23.8	27.6	29.3	30.3
Okayama	17.0	19.8	23.5	26.8	27.3	28.2
Hiroshima	16.6	19.7	23.6	26.8	27.3	28.2
Yamaguchi	17.1	20.0	24.0	27.7	28.7	29.4
Tokushima	17.0	19.3	22.6	26.4	28.1	29.3
Kagawa	16.9	19.5	23.2	26.9	27.7	28.5
Ehime	17.1	19.6	23.1	26.8	28.2	29.5
Kochi	17.8	20.3	23.9	27.7	29.0	30.0
Fukuoka	16.0	18.8	22.3	25.8	27.0	27.8
Saga	17.0	19.1	22.1	25.9	28.3	29.6
Nagasaki	17.1	19.2	22.3	26.3	28.9	30.6
Kumamoto	17.5	19.6	22.6	26.4	28.8	30.3
Oita	17.2	19.8	23.4	27.4	29.2	30.2
Miyazaki	17.2	19.4	22.6	26.9	29.5	30.9
Kagoshima	17.7	19.3	22.0	26.0	29.0	30.8
Okinawa	16.1	17.8	20.0	23.1	25.4	27.1
National average	16.7	19.4	22.7	26.1	27.4	28.7

In 2020, the prevalence did not exceed 20% in any prefecture. In 20,205, five prefectures, mainly rural prefectures, are forecast to have rates exceeding 20%, and by 2030, the rate will exceed 20% nationwide. By 2035, the rate will exceed 25% in all but five prefectures. In 2045, all prefectures excluding Tokyo will have a prevalence exceeding 25%, including several prefectures with a prevalence exceeding 30%.

## Discussion

In 2020, no prefecture in Japan had a dementia prevalence exceeding 20%, but the current study results indicated that the rate could exceed 25% in all but one province by 2045. If the growth of dementia in Toyama Prefecture over the past 30 years continues nationwide, it is estimated that one of every 3.5 people older than 65 year in Japan will have dementia in 25 years. The data suggested that the increase in dementia will occur in both rural and urban areas, albeit with a time lag.

To address this problem, nationwide preventive measures are needed to delay the onset of dementia as much as possible. Compared with other countries, such as Europe and the United States, where research on dementia has been conducted since 1980, Japan has been the only country to report an increase in the prevalence of dementia, whereas the other countries have been reported a decrease or stability in the prevalence of dementia [10–12]. This might be due to the fact that Japan has the fastest aging population among developed countries and the longest average life expectancy in the world [13, 14]. As the life expectancy increases, the prevalence rate—calculated by multiplying the incidence rate with the average prevalence period—increases. However, the situation where the population is aging, and average life expectancy is increasing is the same in Europe and the United States. Regarding low educational background—one of the risk factors for dementia, although Japan lagged slightly behind Europe, the United States, and other countries, the education system was reformed in 1947 and its educational environment has improved since the World War II, with compulsory education for 9 years and people graduating at the age of 15 years on average. The impact of low education on dementia is not very different from that observed in Europe and the United States. Thus, what explains the difference in prevalence? One explanation could be other risk factors such as diabetes, stroke, and heart disease. The measures taken in the UK, for example, may provide a clue. In the UK, a nationwide campaign to reduce smoking and salt consumption, which began in 2005, succeeded in reducing the rate of dementia by 30% over 20 years [14]. In Japan, the Comprehensive Strategy for the Promotion of Dementia Measures was adopted in 2015 [15], and various efforts have been made. However, the prevalence of dementia has not yet declined. One reason for this may be that the incidence of stroke, heart disease, and diabetes has not sufficiently decreased to reduce the prevalence of dementia. For example, the number of people strongly suspicious for diabetes increased from 7.4 million in 2002 to 10 million in 2016 [16]. A previous study in the US suggested that although the prevalence of diabetes is increasing, better treatment of diabetes and a decrease in the number of severe cases may have reduced the incidence of dementia [11]. In Japan, it will be important to continue treatment and prevent the development of serious diseases even after the onset of diabetes. In addition, the total number of patients with heart disease increased from 911,000 in 2002 [17] to 1,732,000 in 2017 [18]. To reduce the risk of heart disease, the UK has implemented national projects such as raising the price of a pack of cigarettes to promote smoking cessation since 2005 and setting numerical targets for the salt content of 85 types of food for the food industry to gradually reduce its levels [14]. In Japan, local governments carefully analyze the health status of their residents based on health checkups and medical cost data to prevent the onset and severity of disease in each municipality, reflecting local characteristics. In fact, the number of patients with stroke has decreased nationwide from 1,729,000 in 1996 to 1,179,000 in 2014 [19], and this may be reflected in the decreased incidence of in cerebrovascular dementia in surveys conducted in Hisayama, Fukuoka Prefecture and in Toyama Prefecture [20, 21]. However, measures that require a society-wide approach, such as reducing salt intake and quitting smoking, are being implemented by local governments to fundamentally change residents’ lifestyles, but their effectiveness and continuity may be limited. The efforts of local governments need to be supplemented by a national strategy that takes them one stage further.

Considering future projections of the prevalence of dementia over time, the national average for 2025 was 19.4%, which was similar to the Hisayama study’s projection of one of five people aged 65 and over with dementia. Although the projected rate exceeded 20% in several prefectures, there were no major differences between regions. By 2030, the prevalence of dementia in Japan is forecast to range 20–25%, and by 2035, the projected prevalence of dementia in all but five prefectures ranged 25–30%. In 2045, prevalence is projected to exceed 30% in 12 prefectures, and the differences between regions became larger. One of the reasons for the regional differences in the prevalence of dementia around 2045 was believed to be related to the estimated future population by sex and age in each prefecture, as the estimated future prevalence of dementia by sex and age was based on that in Toyama Prefecture. The prevalence of dementia is also expected to increase quickly in prefectures in which the proportion of the population aged 80 years or older is quickly increasing. In addition to the increase in the number of people with dementia, the best strategies for managing these patients are unclear. As the number of people with dementia increases, it is necessary to prepare the necessary medical and care systems. However, it has been reported that at present, there are differences in medical and care resources among prefectures [22]. Even if the prevalence of dementia increases quickly in rural prefectures, they can manage this issue if proper medical and nursing care services are provided. However, even in urban areas in which the prevalence of dementia is slowly increasing, cities will be unable to managing the increased prevalence of dementia of medical and nursing care services are delayed. Regarding the development of medical care, each prefecture sets the appropriate number of hospital beds for each medical area based on the Medical Service Law [23]. Regarding long-term care services, according to the long-term care insurance business (support) plan, each prefectural government formulates plans for long-term care insurance facilities, and the municipal governments formulate plans for community-based and other long-term care services based on estimates of the volume of such services [24]. In many cases, the future estimates of service volume in each plan are calculated by multiplying the most recent actual usage rate by the estimated future population. However, caution is required if there is a large difference between the estimates of these local governments and those derived from actual surveys such as the present survey. Facilities for medical and nursing care services for patients with dementia based on local conditions and human resources such as securing care personnel who can treat patients with dementia are required.

In this study, we projected the prevalence of dementia for a period of approximately 25 years. Although this appears to be a long period, considering the period in which the effects of the policies will become apparent, urgent action is needed. In other countries, decades were required before the effects of dementia countermeasures become apparent. Thus, Japan must immediately formulate and implement nationwide plans targeting dementia.

This study had several limitations. First, the future estimates presented in this study have been estimated using only historical dementia prevalence rates and do not incorporate changes in factors that may affect dementia in the future. Future changes in factors include both factors that increase dementia (increase in prevalence of lifestyle-related diseases, increase in average life expectancy) and factors that decrease dementia (decrease in prevalence of lifestyle-related diseases due to early detection and treatment of lifestyle-related diseases, improvement in educational background), which may offset the impact of these factors. Changes in factors related to dementia prevalence in the future should be considered, and projections of dementia prevalence need to be revised accordingly. In addition, this future estimated prevalence is an analysis of dementia as a whole, and it does not provide a distinction between cerebrovascular dementia and Alzheimer’s disease. However, the estimated prevalence of dementia may have been underestimated because of the current situation in which the numbers of patients with heart disease and those suspicious for diabetes who are at risk for Alzheimer’s disease have continued to increase. Second, there is a possibility of a cohort effect in this study. The Japanese education system has undergone changes since the end of World War II, and the rate of advancement in higher education has increased significantly over the past few decades. The people with 9 years of compulsory education reached the age of 65 years around the year 2000, and by the time the 2014 survey was conducted, approximately under the age of 80 years had more than 9 years of education. The number of people with less education will decrease in the future, and the risk of dementia because of less education may decrease. Therefore, the growth in the prevalence of dementia may decline. Third, the future estimates presented in this study were calculated using the prevalence rate in Toyama Prefecture, which may not always match the actual situation in each region. However, the prevalence of dementia in surveys conducted in several local cities in Japan around 2010 was generally around 15% [25], and that in the survey in Toyama Prefecture was comparable. Additionally, there was no significant difference compared with the prevalence by sex and age group in the Hisayama study [1]. The use of the prevalence rate in Toyama Prefecture for other 46 prefectures is a limitation of this study. However, this was not significantly different from other studies, and it did not apparently distort the results significantly. Despite these limitations, there have been few randomly selected surveys of dementia in all prefectures accompanied by psychiatric diagnoses, and it is significant that we were able to estimate the rate over the next 25 years while showing the disparities among prefectures to permit the future development of dementia policies nationwide.

## Conclusions

In Japan, it was predicted that the prevalence of dementia among people aged 65 years and over would exceed 25% nationwide, including metropolitan areas, with a time lag from rural areas over the next 25 years. Preventive measures must be planned and implemented to address the critical increase in the rate of dementia.

## Data Availability

The datasets analyzed during the current study are available from the corresponding author on reasonable request.

## Abbreviation

HDS-R: Hasegawa Dementia Scale-Revised
